# Reusing waste biomass in crop protection—Calcinated oyster shell powder enhances rhizospheric microbial-mediated suppression of root-knot nematodes

**DOI:** 10.3389/fmicb.2025.1625653

**Published:** 2025-08-25

**Authors:** Qipeng Jiang, Jiamin Yu, Yong Wang, Jinfeng Wang, Lianqiang Jiang, Shiping Guo, Yu Qian, Xiangwen Yu, Dongyang Liu, Daojiang Xi, Quan Deng, Wei Ding, Shili Li

**Affiliations:** ^1^College of Plant Protection, Southwest University, Chongqing, China; ^2^Sichuan Branch of China National Tobacco Corporation, Chengdu, China; ^3^Liangshan Prefecture Branch of Sichuan Tobacco Corporation, Xichang, China

**Keywords:** oyster shell powder, root-knot nematode, *Meloidogyne incognita*, microbial community, soil amendments

## Abstract

Root-knot nematodes (RKNs), particularly *Meloidogyne incognita*, are one of the most destructive plant-parasitic nematodes (PPNs) affecting crop production worldwide. Previous earlier study revealed that calcinated oyster shell powder (OSP) possessed excellent suppression of tobacco RKN disease. However, the suppression mechanism of OSP against RKNs still remains unrevealed. Hence, this study aimed to clarify the suppression mechanism of oyster shell powder against RKNs by pot experiments and high-throughput sequencing. The results showed that calcinated OSP reduced over 38% of the tobacco root-knot index by inhibiting the migration of second-stage juveniles of *Meloidogyne incognita* (J2) in soil. Furthermore, calcinated OSP reduced J2 density by 43.69% in the tobacco rhizosphere, and significantly increased soil pH by 0.68; moreover, it increased the contents of soil exchangeable calcium (ExchCa) and exchangeable magnesium (ExchMg) by over 50%. Meanwhile, soil properties, including ExchMg, ExchCa, and pH, enhanced microbial-mediated suppression of J2. Specifically, some taxa within Proteobacteria- and Gemmatimonadota-dominated microbial communities and fungal richness may contribute to suppression of RKNs. Conversely, some taxa within Chloroflexi- and Acidobacteriota-dominated microbial communities may be involved in RKNs' prosperity. Our study suggests that reusing waste oyster shell powder as an innovative antagonist against RKNs presents promising avenues for nature-based PPN management strategies, and would generate significant economic value and social impact in plant protection.

## 1 Introduction

Root-knot nematodes (RKNs) of the genus *Meloidogyne* are among the most destructive plant-parasitic nematodes (PPNs) (Sijmons, 1993). Among the genus *Meloidogyne, Meloidogyne incognita* (*M. incognita*) causes substantial economic losses to crop production worldwide, especially in Solanaceae crop production, such as tomato (El-Sappah et al., 2019; Du et al., 2022), tobacco (Cao et al., 2023; Xu et al., 2023) and eggplant (Khan and Siddiqui, 2017; Zhou et al., 2019). This obligate, soil-borne endoparasite aggregates around the plant root surface, invades the roots, and establishes feeding sites, which hinder nutrient and water uptake, ultimately causing stunted plant growth. Moreover, infection of RKNs causes wounds in the plant roots, which favors further infection of other soil-borne pathogens, leading to complex plant diseases (Parkunan et al., 2016; Kyndt et al., 2017; Zhang Y. et al., 2020). Nowadays, the application of chemical nematicides is still the major effective approach to RKN management in agriculture (Liu et al., 2020). However, considering the safety and environmental pollution, several chemical nematicides have been banned or limited in agricultural use due to their negative impacts on environmental and human health (Ntalli and Caboni, 2012). Thus, there is an increased demand for nature-based solutions to manage RKNs.

Oyster shell is a freely available seafood waste; however, it is also a good calcium-enriched natural product and soil conditioner alternative (Lu et al., 2021). In recent years, oyster shell is widely reused as a waste bioresource. Calcinated oyster shell powder is alkaline and contains abundant calcium ions, making it an economical product in crop production and soil amelioration. Recent research studies have proved that oyster shell powder plays a positive role in increasing crop production (Zhang et al., 2025; He et al., 2021), alleviating soil compaction and acidification (Shen et al., 2018; Lee et al., 2020), improving the fertility of soil (Hong et al., 2021; Yang et al., 2022), and reducing environmental pollution (Lu et al., 2021; Yang H. et al., 2023; Li et al., 2025). Moreover, the application of oyster shell powder to the soil has the potential to suppress soil-borne diseases. (Shen et al. 2018) found that the oyster shell powder addition to soil could control tobacco bacterial wilt by alleviating soil acidification and regulating the composition of the soil bacterial community. (Martial et al. 2023) proved that heat-treated oyster shell powder could improve the defense of *Theobroma cocoa* against *Phytophthora megakarya* by inducing the priming defense system and stimulating the agronomic growth of seedling plants. Nevertheless, there are rare studies focused on the effect of oyster shell powder on RKNs or PPNs. Remarkably, in a previous study, we found that calcinated oyster shell powder showed an impressive control effect of tobacco RKN disease, but the suppression mechanism of oyster shell powder against RKNs still remains unrevealed.

Generally, RKNs' migration, aggregation, and invasion around the plant root surface were affected by diverse factors, such as moisture (Zheng et al., 2023; Frankenstein et al., 2024), root exudates (Ngala et al., 2021; Perry, 2024) and soil microbiome (Lu et al., 2014; Elhadyl et al., 2017). In recent years, culture-independent high-throughput sequencing has greatly expanded the repertoire of plant-associated microbiomes and their roles in plant disease (Liu et al., 2016; Zhang S. et al., 2020; Du et al., 2024), allowing us to explore the complex interaction between pathogen and other microbiomes in the disease process, and reveal the microbial-mediated mechanism of disease outbreak. Although precise and comprehensive studies have unveiled the significant role of rhizospheric microbes in in suppression of RKNs (Jin et al., 2021; Antil et al., 2023), for example, (Cao et al. 2023) observed that RKN-infected tobacco exhibited a richer and more diverse rhizosphere soil bacterial community compared to healthy tobacco. The mechanisms by which oyster shell powder works in the RKN suppression mechanism are still limited, considering the multiple effects of oyster shell powder on soil properties, microbial community, plant growth, and disease control.

The objective of this study was to clarify the suppression mechanism of oyster shell powder against RKNs. The efficiency of oyster shell powder on *M. incognita* control was confirmed in growth tobacco and field experiments. Then, to explore the mechanism by which oyster shell powder suppressed *Meloidogyne incognita*, we used culture-independent high-throughput sequencing to characterize the bacterial and fungal communities in the tobacco rhizosphere. Additionally, the effect of applying oyster shell powder on soil properties was tested. Furthermore, a structural equation model (SEM) was constructed to quantify the effects of soil properties and microbial community on *M. incognita* in the tobacco rhizosphere. Our study proposes a nature-based material for RKN disease control, and will generate significant economic value and social impact in plant protection.

## 2 Materials and methods

### 2.1 Pot experiment design

The pot experiment was conducted 1 January 2022 to 25 February 2022 in the College of Plant Protection, Southwest University, Chongqing, China. Soil collected in the field induced with RKNs disease (*Meloidogyne incognita*) was used in the pot experiment. The field is located in the Liangshan Yi Autonomous Prefecture, Sichuan province (26°17′38″N, 102°01′10″E, Elevation: 1892 m), and has been subjected to continuous tobacco cropping for years. The water content of the experimental soil was maintained at 20%. Oyster shell powder (OS), used in the pot experiment, was purchased from Haixinghaizhiyuan Feedstuff Co., Ltd., Bohai New Area, Hebei, China. The physicochemical characteristics of OS are shown in Supplementary Table S1 (Shen et al., 2018). Honghua Dajiyuan (*Nicotiana tabacum* L., susceptible tobacco) was used in the pot experiment, which was bred and cultured for 30 days under identical conditions by the College of Plant Protection, Southwest University.

The OS and soil were mixed with different mass ratios thoroughly to be used in the pot experiment. Four treatments were set, and each treatment had four repetitions, with 30 tobacco plants in each repetition. The four treatments were as follows: (1) 0.1%OS: the mass ratio of OS to soil is 0.1%; (2) 0.2% OS: the mass ratio of OS to soil is 0.2%; (3) 0.4%OS: the mass ratio of OS to soil is 0.4%; (4) CK: blank control, without any treatment. The mixture of soil from different treatments was cultured for 2 weeks under identical conditions before tobacco transplanting. Tobacco was transplanted to the pot and maintained in a greenhouse at 28°C, and substantial root growth typically occurred during the subsequent 10 days.

### 2.2 Field experiment design

The field experiment was conducted from 1 May 2022 to 25 August 2022 in the RKNs disease-induced tobacco field mentioned above. Yunyan87 (*N. tabacum* L., susceptible tobacco) was used in the field experiment, which was bred and cultured under identical conditions by the Liangshan Prefecture Branch of Sichuan Tobacco Corporation. Three types of different and representative nematicides, including oyster shell powder (mentioned above), 3% Avermectin·fosthiazate (0.5% Avermectin and 2.5% Fosthiazate, purchased from Hainan Zhengye Biotechnology Co., Ltd., Chengmai, Hainan, China), and 0.25 billion live spores/g *Verticillium chlamydosporium* (purchased from Yunnan Weitaiyuan Biotechnology Co., Ltd., Qujin, Yunnan, China), were used in the field experiment, which were often used in the fields to control RKN disease.

Four treatments were implemented in the field experiment, and each treatment had three repetitions (plots). Randomized block design and a triplicated plot were used. Each plot with an area of 66.67 m^2^ was planted with 100 plants (4 lines × 25 plants/line), consisting of four 15 m-long rows (the spacing between two adjacent plants in one line is 0.55 m), spaced 1.2 m apart. The four treatments were as follows: (1) OS: Oyster shell powder, 100 g/plant mixed with soil as nest-fertilizers before tobacco transplanting; (2) AS: 3% Avermectin·fosthiazate, 2 g/plant mixed with soil as nest-fertilizers before tobacco transplanting; (3) HB: 0.25 billion live spores/g *V. chlamydosporium*, 2 g/plant, irrigating just after tobacco transplanting; (4) CK: blank control, without any treatment. All fertilizers used in the tobacco field were applied once as the base manure before tobacco transplanting, additionally under the identical guidelines of the Liangshan Prefecture Branch of Sichuan Tobacco Corporation.

### 2.3 Data investigation

To evaluate the effect of OS on root-knot nematode (*M. incognita*) infection, tobacco roots in the pot experiment were cleared and stained with acid fuchsin 10 days post-transplanting (Lee et al., 2012), then photographed under an inverted fluorescence microscope. Root-knot indexes (number of root-knots per gram of tobacco roots) on tobacco roots in the pot experiment were investigated every 10 days within 30 days (Oka et al., 2012). To evaluate the effect of OS on RKN migration, RKNs in the soil of 0.4%OS and CK were photographed under an inverted fluorescence microscope. To evaluate the effect of OS on tobacco growth, tobacco root activities were investigated by colorimetry at the absorbance of 485 nm every 10 days within 30 days in the pot experiment (Fang et al., 2021), and total chlorophyll content of tobacco leaves were investigated by colorimetry at the absorbance of 663 and 645 nm every 10 days within 30 days in the pot experiment (Faisal et al., 2023).

To evaluate the RKN disease progress of different treatments, the RKN disease grade of tobacco plants aboveground was investigated every 3 days since the first symptomatic day in the field experiment. In the last time of disease investigation, plants were uprooted with the whole root ball, and the soil around the root was carefully removed under running tap water, and the root disease grade for each root was assessed. The criteria of disease grade referred to the national standard of the tobacco pest classification survey method of China (Supplementary Table S2) (Fan et al., 2023).

To evaluate the control effect of different treatments on RKN disease degree, disease index, and area under disease progress (AUDPC) were calculated using the following formulas:


$$\begin{array}{l} \text{Disease index}=\sum \frac{N_{i}\;\times \;v_{i}}{N\;\times \;9}\;\times \;100 \end{array}$$

where N_i_ is the number of plants with the respective disease grade, v_i_ is the disease grade (0, 1, 3, 5, 7, and 9), and *N* is the total number of investigated plants (Ali et al., 2022).


$$\begin{array}{l} \text{AUDPC}=\sum \left(\frac{\left(V_{i}\;+\;V_{i-1}\right)}{2}\;\times \;\left(t_{i}\;-\;t_{i-1}\right)\;\right) \end{array}$$

where t_i_ and t_i − 1_ are two closed days of disease investigation, and V_i_ and V_i − 1_ are the disease indices on t_i_ and t_i − 1_, and t_i_ – t_i − 1_ denotes the days of interval between t_i_ and t_i − 1_ (Dyer et al., 2022).

### 2.4 Soil samples and sample test

To investigate the density of RKNs and identify the characteristics of microbial communities in the rhizosphere of treatments, soil samples from bulk soil and tobacco rhizosphere were collected at 0, 20, 80, and 100 days after transplanting in the field experiment, representing different periods of disease progress, and the sampling depth was 10–20 cm. The collection methods of soil samples followed our previous study (Liu et al., 2016). Five individual soil samples from five randomly selected and closed plants/sites were collected and combined into a single sample. Five samples of representative bulk soil from five randomly arranged sites in the experimental field were collected, and three rhizosphere soil samples for each treatment (three repetitions/plots) were collected. Each soil sample was separated into three even samples (Samples A, B, and C). All Samples were transported to Southwest University, Chongqing, for further testing within 24 h. Sample A was stored at −20°C for DNA extraction, while Samples B and C were kept at indoor temperature (24°C) to determine the density of RKNs and soil properties.

The density of second-stage juveniles of *M. incognita* J2/J2 of each soil sample at 0, 20, 80, and 100 days after transplanting was determined. Hatched J2 in the soil samples were isolated according to the Baermann funnel method, and the population was counted using a stage micrometer under a Nikon microscope (Tokyo, Japan). The density of J2 was converted to numbers per 100 g dry soil (Khan et al., 2024).

The soil properties of the soil sample were measured at 20 and 100 days after transplanting. Soil pH, organic matter (OM), available nitrogen (AvailN), available phosphorus (AvailP), available potassium (AvailK), exchangeable calcium (ExchCa), and exchangeable magnesium (ExchMg) were measured using standard methods in the soil analysis laboratory of Southwest University, China. In particular, soil pH was measured using a pH electrode (Metter-Toledo™; SevenMulti, Switzerland) at a soil:water ratio of 1:2.5 (w/v). OM was determined using the Kjeldahl digestion method (Mariyappillai and Arumugam, 2021) and the dichromate oxidation method (Hernández et al., 2023), respectively. AvailP was extracted with 0.025 mol/L HCl + 0.03 mol/L NH_4_F and measured by a visible spectrophotometer (Yin et al., 2021). AvailK, ExchCa, and ExchMg were determined in ammonium acetate extracts by flame photometry (Shen et al., 2024).

### 2.5 Sequencing

Microbial DNA from soil samples was extracted using the FastDNA Spin Kit (MP Biomedicals, United States kits) following the standard protocol. The extracted microbial DNA was stored at −80°C. The amplification and purification of soil microbial DNA were conducted, adhering to the methods described in our previous study (Liu et al., 2022). ITS1F (5′-CTTGGTCATTTAGAGGAAGTAA-3′) and ITS2 (5′-GCTGCGTTCTTCATCGATGC-3′) were used to amplify the ITS1 region of the fungal ITS gene. The 515F (5′-GTGCCAGCMGCCGCGG-3′) and 806R (5′-GGACTACHVGGGTWTCTAAT-3′) primers were used to amplify the V4 region of bacterial 16S rDNA gene.

The sequencing of 16S ribosomal RNA (rRNA) and ITS gene fragments was conducted by Shanghai Majorbio Co., Ltd., China. using the Illumina MiSeqPE250 platform (https://cloud.majorbio.com). The quality control and annotation of the raw sequencing data were adhering to the methods described in our previous study (Liu et al., 2022). Amplicon sequence variants (ASVs) were assigned to taxa at the phylum, class, order, family, and genus level using QIIME2 (https://library.qiime2.org). The Silva138 database was used in the taxonomic assignments of bacterial and archaeal ASVs, and the Unite 8.0 database was used in the taxonomic assignment of fungal reads.

### 2.6 Data analyses

Microbial Alpha diversity and Beta diversity analysis were performed using Majorbio Cloud Platform (http://www.majorbio.com). Specifically, Alpha diversity indices of bacterial and fungal communities, including Chao1, Shannon, and Shannon evenness index, were calculated based on Faith's phylogenetic metric at the ASV level. Principal Coordinates Analysis (PCoA) was investigated based on the Bray–Curtis distance according to the phylogenetic tree to determine the dissimilarity of the microbial communities. To determine the interaction between bacteria and fungi in the tobacco rhizosphere, the underlying co-occurrence network among bacteria and fungi was depicted at the ASV level (relative abundance above 0.01%) through network analysis on the Molecular Ecological Network analysis pipeline (MEAN, http://ieg4.rccc.ou.edu/mena/login.cgi) and Grephi software (Sun et al., 2023). ASVs represented in more than 50% of the samples were reserved, and data filtering was performed prior to avoid zero values that could result in spurious correlations (Zhao et al., 2019). Structural equation models (SEM) were constructed in IBM Statistical Package for the Social Sciences (SPSS) and Analysis of Moment Structures (AMOS) software to determine the direct effects of OS and bacterial and fungal communities on RKNs. All statistical significances were assessed by ANOVA in SPSS (Statistical Product Service Solutions). Linear regression analysis was executed in SPSS to evaluate the correlation between variates. Other figures were plotted in Origin software.

## 3 Results

### 3.1 Effects of oyster shell powder on tobacco root-knot nematode infection in pot experiments

**Figure 1:**
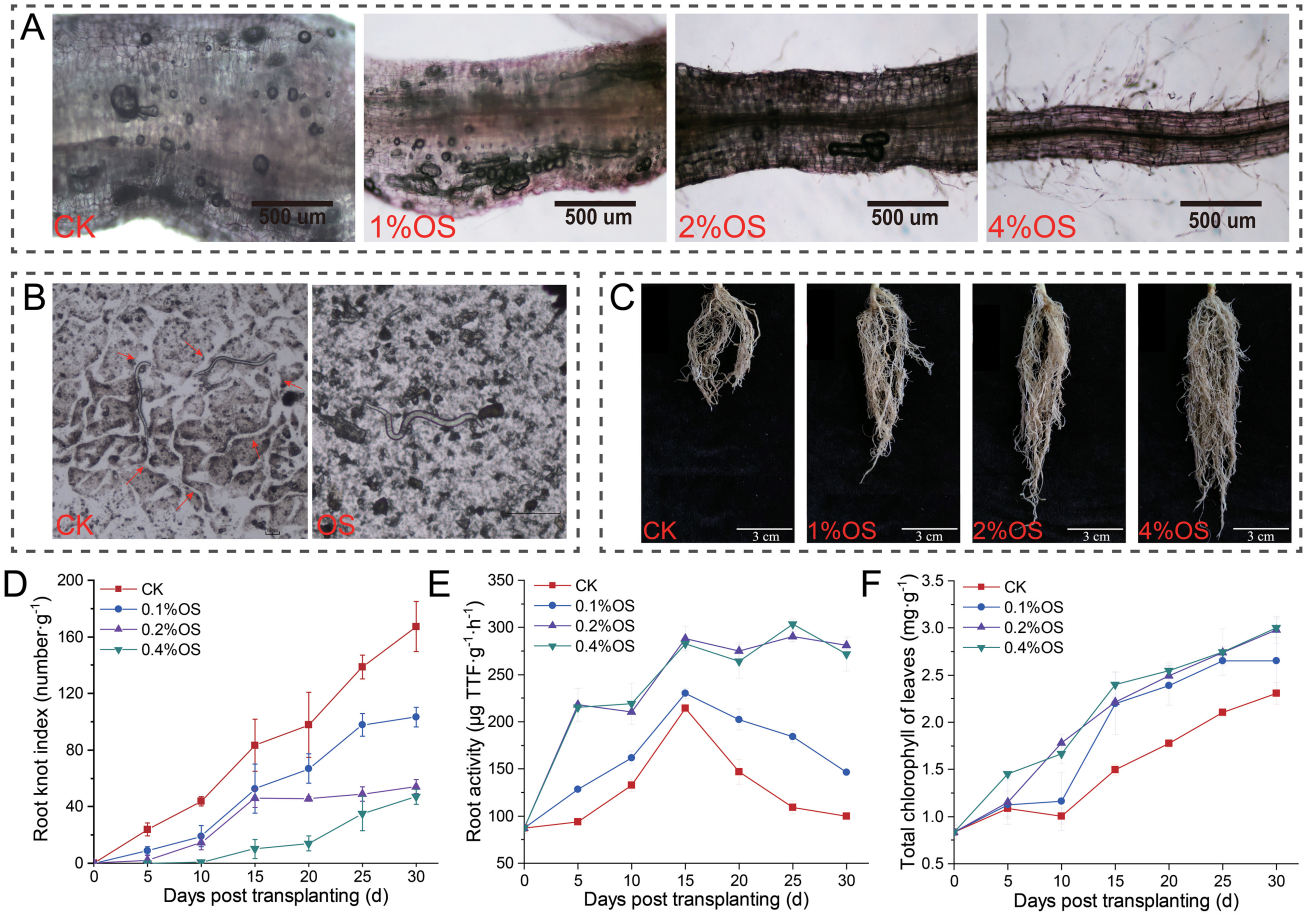
Effects of oyster shell powder on root-knot nematode infection of tobacco. **(A)** Root-knot nematode infection of tobacco roots 10 days post-transplanting. **(B)** Effects of 0.4% oyster shell powder (OS) on migration of root-knot nematode in soil. **(C)** Tobacco root growth 30 days post-transplanting. **(D)** Root-knot indexes of tobacco roots. **(E)** Tobacco root activities. **(F)** Total chlorophyll content of tobacco leaves. 0.1% OS, 0.2% OS, and 0.4% OS represent treatments with different contents of oyster shell powder (OS); CK, blank control.

Effects of oyster shell powder (OS) treatment on root-knot nematodes (RKNs) infection of tobacco roots were investigated in pot experiments (Figure 1), and the results showed that OS treatment presented remarkable suppression on RKNs' infection and migration in soils, and promoted tobacco growth and root activity. Specifically, the giant cells and *M. incognita* J2 (J2) in tobacco roots significantly decreased with the increase of the content of OS in soils (Figure 1A), and migration of J2 in soils mixed with 0.4% OS was significantly confined compared to CK (Figure 1B, Supplementary Figure S1). Meanwhile, significant growth promotion of tobacco root after treatment of OS was observed, and the root growth was positively related to the content of OS in soils (Figure 1C). Tobacco root-knot indexes, root activities, and total chlorophyll content of tobacco leaves were tested to evaluate the effects of oyster shell powder on tobacco under root-knot nematode infection. Root-knot indexes of tobacco roots significantly decreased by 38%−71% (*p* < 0.001) after treatment of OS, and the root-knot indexes were negatively related to the content of OS in soils (Figure 1D). Tobacco root activities were improved by 46−172% (*p* < 0.01) after treatment of OS. Remarkably, the root activities of treatments of 0.2% OS and 0.4% OS still remained at a high level after 15 days post-transplanting, which were significantly different from treatments of CK and 0.1% OS (Figure 1E). In addition, total chlorophyll content of tobacco leaves of 0.2% OS and 0.4% OS were significantly improved over 29% (*p* < 0.01) compared to CK (Figure 1F).

### 3.2 Effects of oyster shell powder on RKN disease and soil properties in field experiments

**Figure 2:**
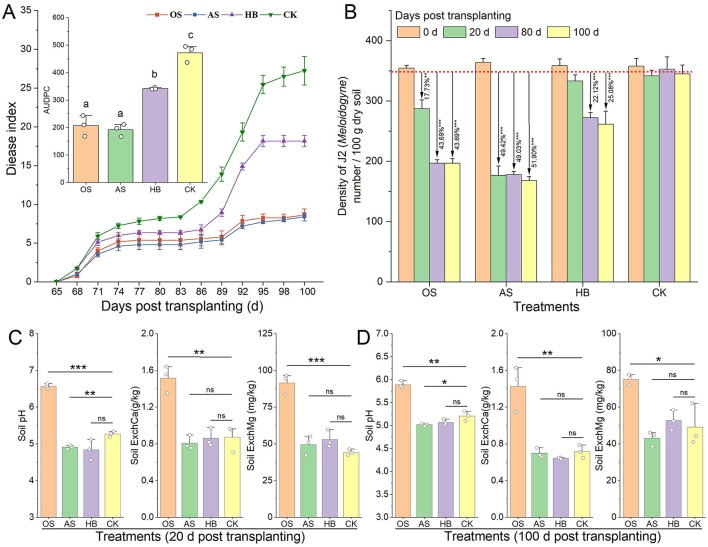
Effects of soil acidification amendment on root-knot nematode disease and soil properties. **(A)** Disease index curve of tobacco root-knot nematode disease and AUDPC. **(B)** Density of *Meloidogyne incognita* J2 in tobacco rhizosphere. **(C)** Soil properties in 20 days post-transplanting. **(D)** Soil properties in 100 days post-transplanting. OS, treatment with oyster shell powder; AS, treatment with avermectin·fosthiazate; HB, treatment with *Verticillium chlamydosporium*; CK, blank control. ExchCa, exchangeable calcium; ExchMg, exchangeable magnesium. ns and ^*^ on the bars marked the significance of difference, ns: *p* > 0.05, ^*^p < 0.05, ^**^p < 0.01, ^***^p < 0.001.

Disease indices of RKN disease of different treatments were investigated in field experiments. Treatments of OS, AS, and HB significantly alleviated tobacco RKN disease in the RKN-induced field, whose disease indexes of were lower than CK within 100 d after tobacco transplanting, and the AUDPC of which (area under disease process curve) were 56.13% (*p* < 0.01), 59.30% (*p* < 0.001) and 27.58% (*p* < 0.01) lower than that of CK (Figure 2A). Additionally, treatments of OS, AS, and HB effectively promote the growth of tobacco in the field (Supplementary Table S1). Density of J2 in tobacco rhizosphere of different treatments within 100 days after tobacco transplanting were investigated (Figure 2B) and the result showed that OS possessed a delayed but durable suppression effect on J2 in the soils, whose density of J2 in 20 days, 80 and 100 days post-transplanting were 15.90% (*p* < 0.01), 43.00% (*p* < 0.001) and 44.23% (*p* < 0.001) lower than that of CK. Nevertheless, the suppression effect of AS on J2 was rapid and durable, with the density of J2 being 49% (*p* < 0.001) lower than that of CK. In addition, the suppression effect of HB on J2 was delayed and limited, whose density of J2 in 80 d and 100 d decreased by 22.12% (*p* < 0.001) and 25.05% (*p* < 0.001) compared to CK.

OS treatment did not change the content of soil available nitrogen (AvailN), available phosphorus (AvailP), available potassium (AvailK), and organic matter (OM) within 100 d post treatment (Supplementary Table S2, *P* > 0.05). Soil properties of different treatments were tested to reveal the effects of OS on soil properties. A rapid and durable improvement of soil pH, content of exchangeable calcium (ExchCa), and exchangeable magnesium (ExchMg), was tested after soil amending by OS (Figures 2C, D). pH of OS increased by 0.68 (*p* < 0.01), and the content of ExchCa and ExchMg of OS increased by 99.13% (*p* < 0.01) and 52.82% (*p* < 0.05) compared to CK in 100 d post-transplanting.

### 3.3 Soil microbial community composition and driving effectors

**Figure 3:**
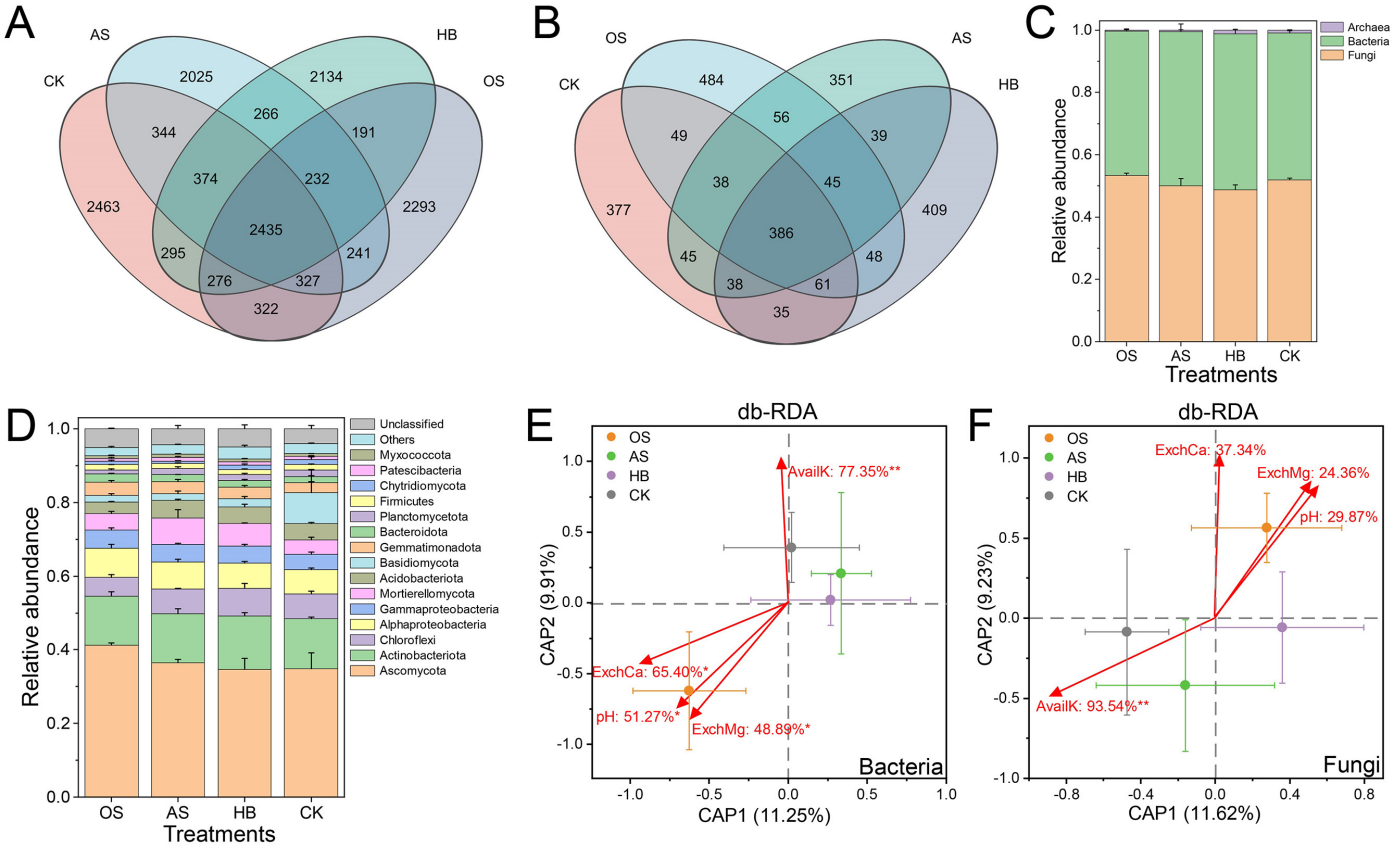
Soil microbial community composition and its driving effectors. **(A)** Venn diagram of bacterial ASVs of different treatments. **(B)** Venn diagram of fungal ASVs of different treatments. **(C)** Community composition at the kingdom level. **(D)** Bacterial-fungal community composition on the phylum level. **(E)** Distance-based redundancy analysis (db-RDA) between soil bacterial communities and soil properties. **(F)** db-RDA between soil fungal communities and soil properties. Every dot was the average of a treatment. Bars on the dots were the standard error (SE) of a treatment. Soil properties were marked with red arrows. Texts on the arrows (soil properties) were the proportion of explained variance in bacterial or fungal communities. *Followed the texts marked the significance of correlation, *p < 0.05, **p < 0.01. OS, treatment with oyster shell powder; AS, treatment with 3% avermectin·fosthiazate; HB, treatment with *Verticillium chlamydosporium*; CK, blank control. pH, Soil pH; AvailK, available potassium; ExchCa, exchangeable calcium; ExchMg, exchangeable magnesium.

Differences in bacterial and fungal community composition at the ASV level were detected. A total of 3,518,268 reads with an average read length of 256 bp were detected from 12 soil samples through 16S ribosomal DNA (rDNA) high-throughput sequencing analysis, clustered into 20,715 ASVs, only 2,435 of which were shared by all treatments, and the proportion of unique bacterial ASVs of OS, AS, HB and CK were 36.30, 32.43, 34.40, and 36.03% (Figure 3A). A total of 4,228,660 reads with an average read length of 238 bp were detected through ITS high-throughput sequencing analysis, clustered into 3,472 ASVs, only 386 of which were shared by all treatments, and the proportion of unique fungal ASVs of OS, AS, HB and CK were 41.47, 35.17, 38.55, and 36.64% (Figure 3B). Relative abundance (RA) of total fungi increased after treatment of OS, and RA of OS, AS, HB and CK in the bacterial-fungal community were 53.35, 50.08, 48.71 and 51.90%, and RA of total bacteria of OS, AS, HB and CK were 46.27, 49.44, 50.09, and 47.22% (Figure 3C). All ASVs were assigned to 12 fungal phyla, 38 bacterial phyla, and 4 archaeal phyla. The most abundant phylum was Ascomycota, whose RA were over 34%, and the RA of Ascomycota of OS were higher than that of other groups (Figure 3D).

The Chao1 index, Shannon index, and Shannon evenness index were calculated to evaluate the richness, diversity, and evenness of soil bacterial and fungal communities (Table 1). Results showed that treatments with OS and HB increased the richness, diversity, and evenness of the soil fungal community, and decreased the richness and diversity of the soil bacterial community. Chao1 index and Shannon index of fungal communities of OS were higher than those of CK over 13.65% (*p* < 0.05) and 7.77% (*p* > 0.05), meanwhile, Chao1 index and Shannon index of bacterial communities of treatments were lower than that of CK (*p* > 0.05).

**Table 1:** Alpha diversity of bacterial and fungal communities of soil samples.

**Microbiome type**	**Treatment**	**Chao1 index**	**Shannon index**	**Shannon evenness index**
Bacteria	OS	3,266.67 ± 22.24a	6.83 ± 0.04a	0.84 ± 0.01a
	AS	3,331.67 ± 46.41a	6.89 ± 0.03a	0.85 ± 0.00a
	HB	3,256.00 ± 119.51a	6.79 ± 0.11a	0.84 ± 0.01a
	CK	3,393.00 ± 66.89a	6.91 ± 0.02a	0.85 ± 0.00a
Fungi	OS	602.33 ± 12.55a	4.16 ± 0.12a	0.65 ± 0.02a
	AS	526.67 ± 5.36b	3.99 ± 0.10a	0.64 ± 0.02a
	HB	544.00 ± 20.82b	4.10 ± 0.09a	0.65 ± 0.01a
	CK	530.00 ± 21.78b	3.86 ± 0.29a	0.61 ± 0.04a

Letters followed the numbers (mean ± SE, n = 3) indicated statistically significant difference (*p* < 0.05, Student–Newman–Keuls). OS, treatment with oyster shell powder; AS, treatment with Avermectin·fosthiazate; HB, treatment with Verticillium chlamydosporium; CK, blank control.

To reveal the key soil properties responsible for the variance of microbial communities, distance-based redundancy analysis (db-RDA) was employed. Results showed that 36.86% of variance in bacterial communities could be explained (*p* < 0.05) by soil AvailK (77.35%, *p* < 0.01), ExchCa (65.40%, *p* < 0.05), pH (51.27%, *p* < 0.05), and ExchMg (48.89%, *p* < 0.05) (Figure 3E). 36.01% of variance in fungal communities could be explained (*p* < 0.05) by soil AvailK (93.54%, *p* < 0.01), ExchCa (37.34%, *p* > 0.05), pH (29.87%, *p* > 0.05), and ExchMg (24.36%, *P* > 0.05) (Figure 3F). Our results indicated that soil AvailK, ExchCa, pH, and ExchMg may play crucial roles in affecting the assembly of bacterial and fungal communities, specifically, soil ExchCa, pH, and ExchMg were the most possible and positive soil properties driving the assembly of bacterial and fungal communities of OS.

### 3.4 Key microbiomes in the fungal-bacterial co-occurrence network regulated by soil properties

**Figure 4:**
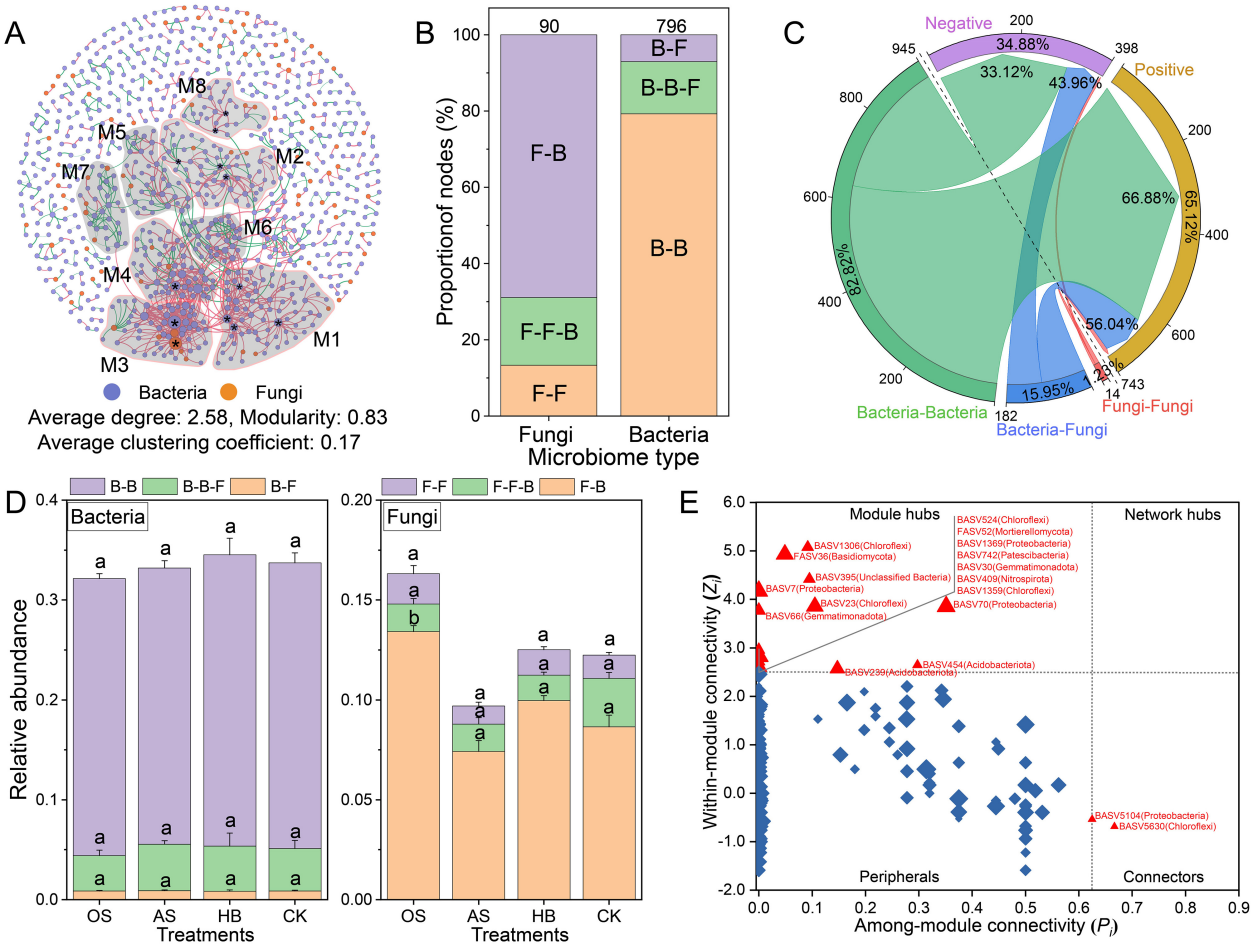
Interaction of fungal-bacterial community. **(A)** The co-occurrence network of bacterial-fungal communities at the ASV level. The node in the co-occurrence network is a bacterial (color-coded in blue) or fungal (color-coded in orange) ASV. Modules are delineated with gray areas and labeled with M1-8, representing modules with 20 or more nodes. The edge between two nodes stands for a significant correlation (r ≥ 0.6, *p* < 0.05). Positive correlations are colored in pink, whereas negative correlations are colored in green. *The identified module hubs (*Z*_*i*_ ≥ 2.50, *p*_*i*_ ≤ 0.62). **(B)** The composition of node types with different connections to other nodes. F-F: fungi only connected with fungi, F-F-B: fungi connected with fungi and bacteria, F-B: fungi only connected with bacteria, B-B: bacteria only connected with bacteria, B-B-F: bacteria connected with bacteria and fungi, B-F: bacteria only connected with fungi. Numbers over the charts were the total number of nodes. **(C)** The composition of edge types with different correlations. **(D)** The RA of bacterial (left) and fungal (right) nodes with different connection types in different treatments. **(E)** Classification of nodes to identify putative keystone ASVs within the co-occurrence networks. Each symbol represents an ASV from the network selected for detailed module analysis. The size of each symbol was related to the RA of the ASV, and the crucial nodes were marked out on the symbols. OS, treatment with oyster shell powder; AS, treatment with avermectin·fosthiazate; HB, treatment with *Verticillium chlamydosporium*; CK, blank control.

A co-occurrence network with 796 representative bacterial ASVs and 90 fungal ASVs was constructed to reveal the interaction patterns between bacterial and fungal communities. The result showed that the co-occurrence network of the bacterial–fungal community was highly modularized (modularity = 0.83), and eight modules (M1–M8) were identified (number of nodes ≥ 20, Figure 4A). The composition of nodes with different connection types in the co-occurrence network was analyzed (Figure 4B), and the result showed that bacteria tend to interact more with bacteria (79.27%), but fungi trend to interact more with bacteria, specifically, 68.89% of fungi in the co-occurrence network were only interact with bacteria. The composition of interaction types (edges) in the co-occurrence network suggested that taxa tended to co-occur (positive correlations, pink lines) rather than co-exclude (negative correlations, green lines), with 65.12% of edges were positive correlations. Approximately 56.04% of interaction between bacteria and fungi were positive (Figure 4C), additionally, the RA of fungi only interacted with bacteria, including FASV983 (Ascomycota), FASV929 (Ascomycota), and FASV421 (Ascomycota), were significantly higher in OS than that of other treatments (Figure 4D), remarkably, all of them were located in M2. Furthermore, 16 module hubs (*Z*_*i*_ ≥ 2.50, *p*_*i*_ ≤ 0.62) were identified as keystone taxa due to their important roles in network topology (Figure 4E), 13 of which were classified in the four modules, including M1 (four hubs), M2 (three hubs), M3 (three hubs), M4 (one hub) and M8 (two hubs).

**Figure 5:**
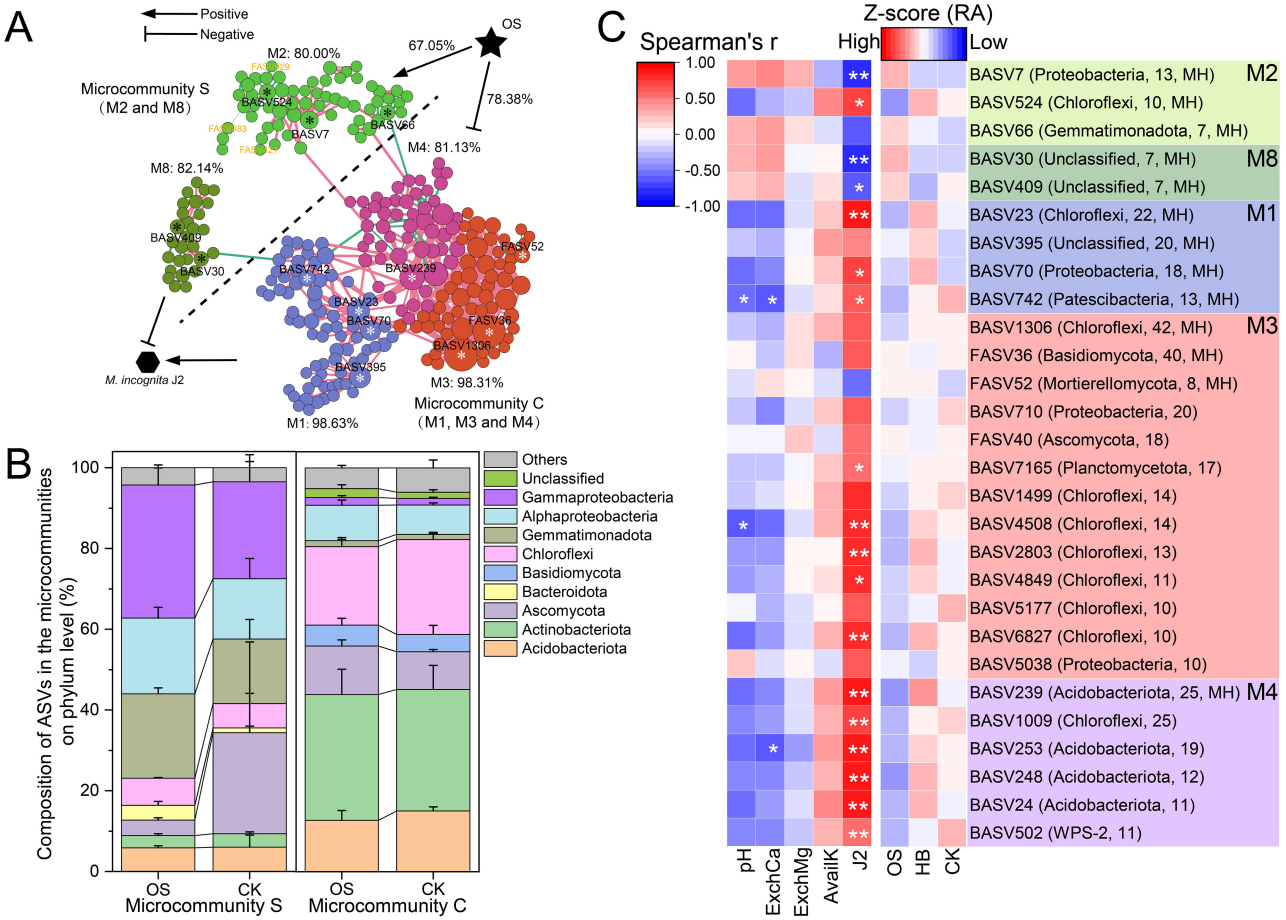
The key microbiomes related to RKNs. **(A)** Modules possessing module hubs (M1, M2, M3, M4, and M8) in the co-occurrence network are depicted. Each node represents an ASV. M2 and M8 are identified as a microcommunity S, which was negatively related to J2, while M1, M3, and M4 are identified as a microcommunity C, which was positively related to J2. Numbers followed by modules are the proportion of nodes in the module negatively or positively related to J2. Stars on the nodes mark key ASVs in the module. ASVs marked with white stars are positively related to J2 and are negatively regulated by OS. ASVs marked with black stars are negatively related to J2 and are positively regulated by OS. Numbers on the connection of OS and modules are the proportion of nodes positively or negatively regulated by OS. **(B)** Composition of the ASVs in the microcommunity S and microcommunity C on the phylum level. **(C)** RA of key ASVs in the modules among different treatments and their correlation with J2 and soil properties. Labels following the ASV numbers indicated the classified phylum and degree of ASV in the whole co-occurrence network. OS, treatment with oyster shell powder; AS, treatment with 3% avermectin·fosthiazate; HB, treatment with *Verticillium chlamydosporium*; CK, blank control. pH, Soil pH; AvailK, available potassium; ExchCa, exchangeable calcium; ExchMg, exchangeable magnesium. ^*^ marked the significance of correlation, ^*^p < 0.05, ^**^p < 0.01.

To explore key ASVs responsive to J2, M1, M2, M3, M4, and M8 of the co-occurrence network was depicted separately. The results showed that 80.00% of ASVs in M2 were negative related to J2 density, and 71.67% of the ASVs in M2 were positively regulated by OS. While 98.63, 98.31, and 81.13%of ASVs in M1, M3, and M4 were positively related to J2 density, and 78.38% of all ASVs in M1, M3, and M4 were negatively regulated by OS (Figure 5A). Considering the close relationship between the five representative modules, J2, M2, and M8 were defined as microcommunity S, which could suppress J2 in the tobacco rhizosphere. Meanwhile, M1, M3, and M4 were defined as microcommunity C, which could benefit J2 in the tobacco rhizosphere. Additionally, microcommunity S possessed a quite different composition of ASVs compared to microcommunity C. Specifically, Gammaproteobacteria (28.09%), Gemmatimonadota (18.67%), and Alphaproteobacteria (16.74%) were the most abundant taxa in microcommunity S, however, Actinobacteriota (27.11%), Chloroflexi (22.42%), and Acidobacteriota (14.42%) were the most abundant taxa in microcommunity C (Figure 5B).

RA of Key ASVs (module hubs or dot degree ≥ 10) and their correlation with J2 and soil properties in microcommunity S and microcommunity C were analyzed (Figure 5C). Module hubs in microcommunity S, including BASV7 (Gammaproteobacteria), BASV66 (Gemmatimonadota), BASV30 (Gemmatimonadota), and BASV409 (Nitrospirota), were negatively related to J2, and the RA of them in OS were 30.20, 39.46, 29.13, and 9.29% higher than that of CK. However, module hubs in microcommunity C, including BASV742 (Patescibacteria), BASV239 (Acidobacteriota), BASV23 (Chloroflexi), and BASV70 (Alphaproteobacteria), were positively related with J2, and the RA of them in OS were 75.52, 43.00, 19.97, and 3.99% lower than that of CK. Additionally, 32.67% of the variance in Microcommunity S and microcommunity C could be explained (*p* < 0.05) by soil AvailK (78.71%, *p* < 0.05), ExchMg (31.33%, *p* > 0.05), pH (27.00%, *p* > 0.05), and ExchCa (25.46%, *p* > 0.05) based on db-RDA (Supplementary Figure S1).

### 3.5 Effect of soil properties and key microbiomes on *Meloidogyne incognita*

**Figure 6:**
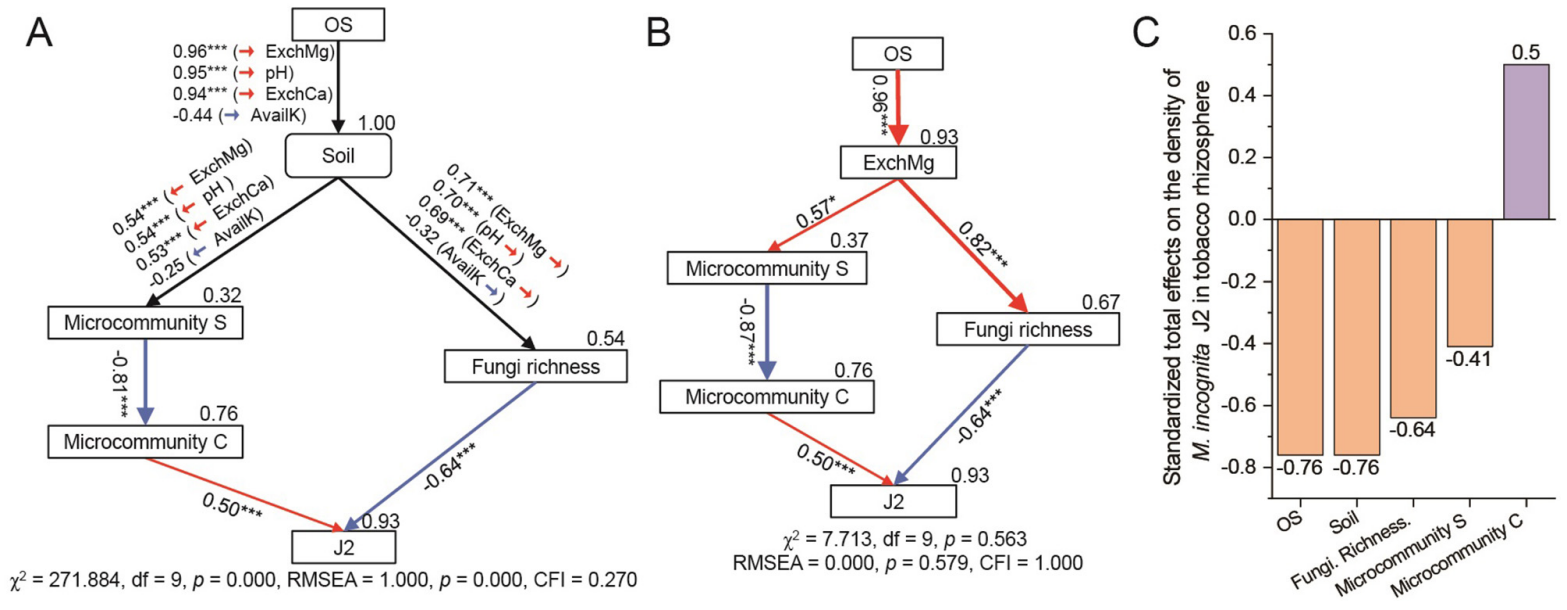
Biotic and abiotic drivers in regulating the density of *Meloidogyne incognita* J2. **(A, B)** The structural equation model (SEM) showing the influences of the OS treatment, soil properties, and microbial profiles on J2. Red arrows represent significant positive pathways, and blue arrows represent significant negative pathways. The width of each arrow is proportional to the strength of the relationship, and numbers near the pathway represent the standardized path coefficients. Bootstrap-based *p*-values for path coefficients are indicated by ***p < 0.001, and *p < 0.05. Numbers on the rectangle represent the proportion of variance explained. χ^2^, chi-square; df, degrees of freedom, df = 9, *p*, probability level; RMSEA, root mean square error of approximation; CFI, comparative fit index. **(C)** Standardized total effects (direct and indirect effects) based on SEM. Soil refers to four soil properties [soil pH, the content of soil AvailK (available potassium), ExchCa (exchangeable calcium) and ExchMg (exchangeable magnesium)]. Microcommunity S and Microcommunity C refer to the average relative abundance of ASVs of the modules. J2: density of second-stage juveniles of *M. incognita*. Fungi richness refers to the Chao1 index of the fungal community based on all fungal ASVs. OS: treatment by oyster shell powder.

To quantify the effects of soil properties and microbial community on J2, a structural equation model (SEM) was then constructed with the presumed relationships among the selected subsets, including soil properties (soil pH, AvailK, ExchCa, and ExchMg), RA of microcommunity S and microcommunity C, the Chao1 index of fungal community (Fungi richness) and J2 density (J2), considering that these selected subsets were least correlated while accounting for multiple drivers simultaneously. The positive and direct effects were maintained. When considering soil properties with pH, AvailK, ExchCa, and ExchMg simultaneously (Figure 6A), the SEM was verified of poor goodness-of-fit [χ^2^ = 271.884, df = 9, *p* = 0.000, root mean square error of approximation (RMSEA) = 1.000, *p* = 0.000, CFI = 0.270], suggesting that soil properties and microbial community characteristic had close relationship with the J2 density, but the SEM need to be modified. Then, A new SEM was constructed only considering one type of soil properties (Figure 6B, Supplementary Figure S2), and the results revealed that a SEM only considering ExchMg as the driver of microbial community was verified of strong goodness of fit (χ^2^ = 7.713, df = 9, *p* = 0.563, RMSEA = 0.000, p = 0.579, CFI = 1.000). Fungi richness and microcommunity C were the most potential and direct factors affecting J2 density in tobacco rhizosphere, and they could explain 93% of the variations in J2 density (Figure 6B), specifically, J2 was negatively affected by Fungi richness (Standardized total effects (STE) = −0.64, *p* < 0.001) and positively affected by microcommunity C (STE = 0.50, *p* < 0.001). Furthermore, Soil ExchMg, positively regulated by OS treatment (STE = 0.96, *p* < 0.001), possessed positive effects on microcommunity S (STE = 0.57, *p* < 0.05) and Fungi richness (STE = 0.82, *p* < 0.001). Additionally, the total effects (direct and indirect) of OS, ExchMg, microcommunity S, microcommunity C, and Fungi richness on J2 were −0.75, −0.78, 0.64, −0.44, and 0.50. Overall, OS promoted the microbial-mediated suppression of J2 by increasing the content of soil ExchMg in the tobacco rhizosphere.

## 4 Discussion

There has been considerable progress in use and development of bioresource as a strategy to manage PPNs (Boelter et al., 2020). (Cole et al. 2021) found that application of biochar could decrease the populations of plant-parasitic nematodes and increase the abundance of predatory nematodes. In this study, we reused the waste oyster shell as a natural bioresource in crop protection and discovered the suppression of calcinated oyster shell powder on the second-stage juvenile of *Meloidogyne incognita* (J2) for the first time. Calcinated oyster shell powder (OSP) reduced tobacco root-knot index and J2 density by inhibiting the migration of J2 and enhancing the microbial-mediated suppression of J2 in the tobacco rhizosphere. Moreover, we unveiled the positive involvement of soil properties, especially soil exchangeable magnesium (ExchMg), in enhancing microbial-mediated suppression of J2, specifically, Chloroflexi and Acidobacteriota-dominating microbes promoted RKNs' prosperity, and Proteobacteria and Gemmatimonadota-dominating microbes and fungal richness contributed to suppression of RKNs in tobacco rhizosphere. Based on these results, calcinated OSP has the potential to be developed as a bioresource to suppress *Meloidogyne incognita*.

China produced about 80% oysters of the world production in 2015 (Bi and Lirong, 2020), and the waste oyster shells were improperly discarded and accumulated, thereby presenting a threat to public health in the past decades (Chung et al., 2021). Therefore, reusing waste oyster shells in crop production presents practical value for natural bioresource recycling, eco-friendly crop protection, and sustainable agriculture. Previous studies have reported that alkaline OSP contains abundant and high dissoluble nutrients, including Ca and Mg (Silva et al., 2019; Yang H. et al., 2023), while this explanation was supported by the results that application of calcinated OSP majorly increased the soil pH, the contents of soil ExchCa and ExchMg in our study. Remarkably, nutrients released by OSP possess several benefits for plant growth promotion and soil microbiome-mediated disease control (Shen et al., 2018). Yang H. et al. (2023) found that OSP promoted the grain weight of rice by ameliorating growth inhibition caused by Cd toxicity. Consistently, agronomic growth promotion of OSP-pretreated tobacco was observed in our field experiment (Supplementary Table S4). (Zhang et al. 2022) revealed that CaO-mediated recruiting of potentially beneficial rhizobacteria enhances disease suppression of bacterial wilt by alleviating soil acidification. Meanwhile, (Yang et al. 2021) discovered that soil bacterial community composition was shaped by exchangeable Mg with short-term application of magnesium fertilizer. (Cao et al. 2023) confirmed that pH level, calcium, magnesium, phosphorus, and iron were identified as key environmental factors influencing the composition of microbial community in tobacco rhizosphere during RKN infection. Besides the direct inhibition on the migration of J2 (Figure 1), our findings suggested the indirect and positive effect of OSP on RKN suppression in the tobacco rhizosphere for the first time, which was mediated by soil ExchMg and microbial community (Figures 2B, C, 6B). The indirect effect of OSP on RKN suppression may be related to rhizospheric microbial community recruited by primed metabolites such as flavonols, brevifolin, and isoflavone compounds (Martial et al., 2023), which need further exploration in the future.

As the rhizosphere is the outpost of RKNs to locate, aggregate, and invade plant roots (Abad et al., 2003; Castagnone-Sereno et al., 2015; Forghani and Hajihassani, 2020), increasing evidence has signified the importance of the rhizosphere microbiome in defending plants from soil-borne pathogens (Dastogeer et al., 2022; Yang K. et al., 2023), therefore, rhizosphere microbiome provide a first line of defense for plants against RKNs (Antil et al., 2023; Banerjee and van der Heijden, 2023). (Cao et al. 2022) observed that the richness and diversity of the fungal community in the healthy group were significantly higher than that of RKN infection, and our finding that J2 density was negatively affected by fungi richness in the SEM was consistent with their result (Figure 6). Notably, some specific fungi are natural enemies of nematodes with direct or indirect effects on nematode inhibition (Soares et al., 2024). The OSP-promoted increase of fungi richness may result in the increase of nematophagous fungi and beneficial microbes, thereby reducing the RKNs' population (Rahman et al., 2024) and plant growth-promoting (Li et al., 2025). Meanwhile, our findings indicated that some taxa within Chloroflexi and Acidobacteriota may be involved in RKN prospering in tobacco rhizosphere; on the contrary, some taxa within Proteobacteria and Gemmatimonadota may be involved in RKN inhibition (Figure 6B). Previous studies have suggested the relationship between the four types of bacterial groups and RKNs. (Jiang et al. 2023) found that nematodes enriched certain soil microbiome groups, including Chloroflexi and Actinobacteria, in a wheat field, and (Su et al. 2022) discovered that Chloroflexi and Acidobacteria dominated the bacterial communities of soil with a higher abundance of nematodes. Additionally, *Ralstonia solanacearum* abundance also correlated positively with Chloroflexi and Acidobacteria abundance, but negatively with Proteobacteria abundance (Zheng X. F. et al., 2019). These studies partly support our identification of Chloroflexi and Acidobacteria as RKN disease-induced microbiomes. There is a rare report about the relationship between Gemmatimonadota and RKNs, but (Li et al. 2022) reported a potential plant growth-promoting function of Gemmatimonadota, as a significant increase in strawberry yield was positively correlated with increases in Gemmatimonadota, and (Mugnai et al. 2024) revealed that Gemmatimonadota could promote both inter- and intra-kingdom interactions of the soil microbial community. These characteristics of Gemmatimonadota partly support our identification of it as a disease-suppressing microbiome. However, the identification of Proteobacteria's relationship with RKNs in previous studies was inconsistent. For example, Zheng F. et al. (2019) found that the diversity of the nematode *Dorylaimus stagnalis* negatively correlated with the abundance of Proteobacteria in rice fields in Ningbo, China, but (Castillo et al. 2017) found that the Alphaproteobacteria positively correlated with *Meloidogyne chitwoodi*, and the Gammaproteobacteria positively correlated with *Pratylenchus neglectus* in potato farms in Colorado. So, the effect of Proteobacteria on RKN disease still needs to be investigated.

This study presented promising avenues of reusing waste oyster shell as an innovative product to control RKNs. Our findings highlighted the microbial-mediated suppression of *Meloidogyne incognita* and the significant regulating role of magnesium ions in the microbial-mediated disease suppression. However, our results about the effect of microcommunities on RKNs and the regulating role of magnesium ions relied on sequencing data and correlation analysis, and we believe that experimental validation, such as field trials or greenhouse experiments demonstrating those direct effects in the future, would further confirm our findings and make our conclusion more robust. Moreover, evaluating the potential control effect of OSP on other types of PPNs would make it more applicable in crop protection.

## 5 Conclusion

In summary, this study put forward the OSP-regulated and microbial-mediated inhibition of tobacco root-knot nematodes for the first time. Calcinated oyster shell powder reduced tobacco root-knot index and *Meloidogyne incognita* (J2) density by inhibiting the migration of J2 and enhancing the microbial-mediated suppression of J2 in the tobacco rhizosphere. Moreover, soil properties, especially soil exchangeable magnesium, exchangeable calcium, and pH, enhanced microbial-mediated suppression of J2. Specifically, some taxa within Proteobacteria and Gemmatimonadota dominated microbial community and fungal richness may contribute to suppression of RKNs, and some taxa within Chloroflexi and Acidobacteriota dominated microbial community may be involved in RKNs' prosperity. However, the interaction mechanism of disease-related microbiome and *Meloidogyne incognita* still needs to be revealed. We believe that reusing waste oyster shell powder as an innovative antagonist against RKNs presents promising avenues for nature-based PPN management strategies.

## Data Availability

The data that support the findings of this study are openly available in the NCBI Sequence Read Archive (SRA) database under accession number PRJNA1080250 for 16S rRNA, and PRJNA1080272 for ITS.
