# Supplementary material for: Reusing waste biomass in crop protection—Calcinated oyster shell powder enhances rhizospheric microbial-mediated suppression of root-knot nematodes
Source: Front Microbiol. 2025 Aug 25;16:1625653. doi: 10.3389/fmicb.2025.1625653 (PMC12414952; doi:10.3389/fmicb.2025.1625653)
Supplement: Supplementary file 1 [file Data_Sheet_1.pdf]

Table S1 Information of the materials used in this study

Materials	Source
Oyster shell powder	Haixinghaizhiyuan Feedstuff Co., Ltd., Bohai New Area, Hebei, China
3% avermectin·fosthiazate (0.5% avermectin and 2.5% fosthiazate)	Hainan Zhengye Biotechnology Co., Ltd. Chengmai, Hainan, China
0.25 billion live spores/g *Verticillium chlamydosporium*	Yunnan Weitaiyuan Biotechnology Co., Ltd., Qujin, Yunnan, China

Table S2 The physiochemical characteristics of the oyster shell powder

**Characteristics**	Plant height (cm)
pH	9.50
$CaCO_3$	98.90%
Protein	0.50%
Magnesium/Mg	2.04 g/kg
Crude fat	0.10%
Iron/Fe	285.00 mg/kg
Potassium/K	214.00 mg/kg
Manganese/Mn	81.00 mg/kg
Phosphorous/P	48.00 mg/kg
Zinc/Zn	2.90 mg/kg

Table S3 The criteria of tobacco RKNs disease grade

The aboveground disease grades: description of tobacco growth and leaf wilting	
Grade 0	Growth normal.
Grade 1	Growth normal, and the margin or tip of leaves are chlorosis but not wilted.
Grade 3	One-fourth to one-third shorter than normal plants, and the margin or tip of a small number of leaves wilted.
Grade 5	One-third to one-half shorter than normal plants, and more than half of leaves have wilted margins and tips or wilted spots.
Grade 7	More than half shorter than normal plants, and the margin and tip of all leaves wilted, or all leaves have wilted spots.
Grade 9	Plants stunted severely, and all leaves wilted.
The root disease grades: description of root and root knot (s)	
Grade 0	Root normal.
Grade 1	A small number of root knots on less than a quarter of the root.
Grade 3	A small number of root knots on a quarter to a third of the roots.
Grade 5	One-third to one-half of the root has a root knot.
Grade 7	More than half of the roots has a root knot, incl. a small number of secondary roots.
Grade 9	All roots, including secondary roots, are covered with root knots.

$$\text{Disease index} = \sum \frac{N_i \times v_i}{N \times 9} \times 100$$

where N_i is the number of plants with the corresponding disease grade, v_i is the disease grade (0, 1, 3, 5, 7 and 9), and *N* is the total number of investigated plants.

$$\text{AUDPC} = \sum \left(\frac{(V_i + V_{i-1})}{2} \times (t_i - t_{i-1}) \right)$$

where t_i and t_{i-1} are two closed days of disease investigation, V_i and V_{i-1} are the disease indices on t_i and t_{i-1}, respectively, and t_i - t_{i-1} is the number of days in the interval between t_i and t_{i-1}.

Table S4 Tobacco agronomic characters 100 d post transplanting of different treatments

Treatments	Plant height (cm)	Stem girth (cm)	Number of productive leaves	Area of maximum leaf (cm^2)
OS	91.76±0.53b	11.27±0.03b	15.83±0.19bc	1512.51±10.63b
AS	92.97±0.45b	11.43±0.03c	16.57±0.16bc	1508.24±12.20c
HB	89.74±0.66a	11.34±0.04bc	15.43±0.18ab	1475.9±10.37ab
CK	86.09±0.63a	11.10±0.03a	14.90±0.19a	1450.63±14.68a

Table S5 Soil properties of different treatments.

Days post transplanting	Treatments	pH	AvailN（mg/kg）	OM（g/kg）	AvailP（mg/kg）	AvailK（mg/kg）	ExchCa（g/kg）	ExchMg（mg/kg）
20 d	OS	6.57±0.04a	42.86±37.03a	16.88±0.66a	22.42±1.02ab	454.44±48.66a	1.52±0.09a	91.53±4.04a
	AS	4.91±0.03c	42.90±40.97a	17.35±0.66a	25.31±1.07a	592.22±82.69a	0.81±0.04b	49.57±3.72b
	HB	4.84±0.16c	41.22±39.83a	17.35±0.16a	17.65±1.07b	639.56±66.08a	0.86±0.06b	53.06±3.43b
	CK	5.27±0.04b	44.50±40.29a	17.92±0.33a	21.59±1.87ab	472.67±48.56a	0.88±0.08b	44.23±1.21b
80 d	OS	5.82±0.04a	46.46±40.36a	22.83±0.67a	23.31±0.26a	186.33±17.48ab	1.52±0.17a	97.89±4.56a
	AS	5.03±0.02c	44.57±39.67a	22.79±0.76a	24.40±0.81a	204.33±3.28a	0.82±0.10b	47.71±2.73b
	HB	5.13±0.05c	47.31±40.41a	22.76±0.36a	22.77±0.31a	161.67±0.88b	0.92±0.01b	49.57±2.06b
	CK	5.29±0.01b	45.73±40.61a	22.09±0.50a	23.16±0.59a	198.00±5.51ab	0.84±0.08b	45.75±2.79b
100 d	OS	5.89±0.05a	42.80±40.32a	20.33±1.22a	25.55±0.28b	191.33±11.61a	1.43±0.14a	75.11±1.67a
	AS	5.02±0.02c	44.20±40.32a	17.07±1.96a	20.67±1.00c	215.33±13.28a	0.70±0.03b	53.14±11.63a
	HB	5.07±0.04bc	44.22±39.73a	21.09±0.41a	21.13±0.42c	207.11±10.62a	0.64±0.01b	52.81±3.17a
	CK	5.21±0.06b	42.55±39.51a	15.87±1.00a	29.38±1.27a	204.00±12.39a	0.72±0.04b	49.15±6.55a

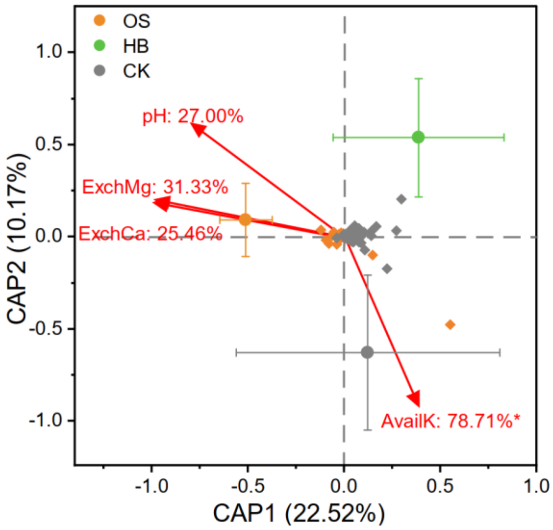

Fig.S1 Driving effectors of the Microcommunities Distance-based redundancy analysis (db-RDA) between the key ASVs and soil properties. Soil properties were marked with red arrows. Texts on the arrows were the proportion explained of variance in bacterial or fungal communities. * followed the texts marked the significance of correlation, *: $p < 0.05$.

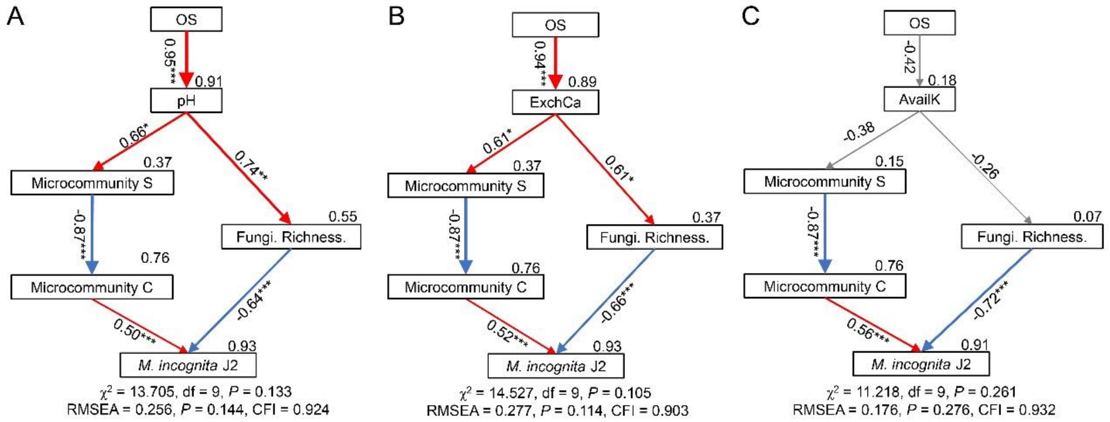

Fig.S2 Biotic and abiotic drivers in regulating the density of *M. incognita* J2. (A)(B) The structural equation model (SEM) showing the influences of the soil pH (A), the content of soil ExchCa (B) and AvailK (C) on the density of *M. incognita* J2. Red arrows represent significant positive pathways, and blue arrows represent significant negative pathways. The width of each arrow is proportional to the strength of the relationship and numbers near the pathway represent the standardized path coefficients. Bootstrap-based *p*-values for path coefficients are indicated by *** when $p < 0.001$, ** when $p < 0.01$ and * when $p < 0.05$. Numbers on the rectangle represents the proportion of variance explained. Microcommunity S and Microcommunity C refers to the average relative abundance of ASVs of the modules. Richness of fungi refers to the Chao1 index of fungal communities based on all fungal ASVs.
